# Evidence of Use of Whole-Body Vibration in Individuals with Metabolic Syndrome: A Systematic Review and Meta-Analysis

**DOI:** 10.3390/ijerph20043765

**Published:** 2023-02-20

**Authors:** Ana Carolina Coelho-Oliveira, Bruno Bessa Monteiro-Oliveira, Raphael Gonçalves de Oliveira, Aline Reis-Silva, Luiz Felipe Ferreira-Souza, Ana Cristina Rodrigues Lacerda, Vanessa A. Mendonça, Alessandro Sartorio, Redha Taiar, Mario Bernardo-Filho, Danúbia Sá-Caputo

**Affiliations:** 1 Programa de Pós-Graduação em Fisiopatologia Clínica e Experimental, Universidade do Estado do Rio de Janeiro, Rio de Janeiro 20551-030, RJ, Brazil; 2 Laboratório de Vibrações Mecânicas e Práticas Integrativas, Departamento de Biofísica e Biometria, Instituto de Biologia Roberto Alcântara Gomes, Policlínica Universitária Piquet Carneiro, Universidade do Estado do Rio de Janeiro, Rio de Janeiro 20950-003, RJ, Brazil; 3 Centro de Ciências da Saúde—Campus Jacarezinho, Universidade Estadual do Norte do Paraná, Jacarezinho 86360-000, PR, Brazil; 4 Programa de Pós-Graduação em Ciências Médicas, Faculdade de Ciências Médicas, Universidade do Estado do Rio de Janeiro, Rio de Janeiro 20550-170, RJ, Brazil; 5 Programa de Pós-Graduação em Reabilitação e Desempenho Funcional, Faculdade de Ciências Biológicas e da Saúde, Universidade Federal dos Vales do Jequitinhonha e Mucuri (UFVJM), Diamantina 39100-000, MG, Brazil; 6 Istituto Auxologico Italiano, Istituto di Ricovero e Cura a Carattere Scientifico (IRCCS), Experimental Laboratory for Auxo-Endocrinological Research, 20145 Milan, Italy; 7 MATériaux et Ingénierie Mécanique (MATIM), Université de Reims Champagne Ardenne, F-51100 Reims, France

**Keywords:** mechanical vibration, metabolic syndrome, quality of life, functionality, pain level, cardiovascular responses, flexibility, body composition, cardiovascular risk

## Abstract

(1) Background: Metabolic syndrome (MSy) is defined by a constellation of interconnected physiological, biochemical, clinical, and metabolic factors that directly increase the risk of cardiovascular disease. This systematic review with meta-analysis was conducted to assess the effects of whole-body vibration exercise (WBVE) in metabolic syndrome (MSy) individuals. (2) Methods: An electronic search in Pubmed, Embase, Scopus, Web of Science, ScienceDirect, PEDro, and CINAHL databases in December 2022 was performed. Data regarding the included studies were extracted. The level of evidence, the methodological quality, and the risk of bias of each selected publication were individually evaluated. (3) Results: Eight studies were included in the systematic review and four studies in the meta-analysis, with a mean methodological quality score on the Physiotherapy Evidence Database (PEDro scale) of 5.6, considered “fair” quality. The qualitative results suggested positive effects of the systemic vibration therapy in relevant outcomes, such as quality of life, functionality, pain level, trunk flexibility, cardiovascular responses (blood pressure and heart rate), neuromuscular activation, range of motion of the knees, rating of perceived exertion, and body composition. The quantitative results, with weighted mean differences, standard mean differences, and 95% confidence intervals (CIs), were calculated. Conclusions: WBVE may be an alternative capable of interfering with physical—mainly for flexibility with weighted mean differences (1.70; 95% CI 0.15, 3.25; *n* = 39)—functional, psychosocial, neuromuscular, emotional parameters, and consequently contribute to improvements in metabolic health and reduce the cardiovascular risk factor in MSy individuals. Nevertheless, further additional studies are required to understand the long-term effects of WBVE on MSy and its complications in a better way. Protocol study registration was as follows: PROSPERO (CRD 42020187319).

## 1. Introduction

In the current worldly conditions, unhealthy lifestyles, including physical inactivity and bad dietary habits, contribute to the development and spread of diseases associated with metabolic commitment, such as metabolic syndrome (MSy) [1]. According to the International Diabetes Federation, MSy is a cluster of metabolic abnormalities that increases the cardiovascular risk, characterized by the presence of abdominal obesity associated with two other factors, such as arterial hypertension, dyslipidemia, and insulin resistance or the presence of Diabetes mellitus type 2 [2]. MSy individuals are largely asymptomatic, and the prevalence is increasing worldwide, representing a serious public health and clinical challenge everywhere. The underlying causes of MSy include overweight and obesity, physical inactivity, genetic factors, and aging, thus contributing to the reduction of functional performance [3], increased stress and, consequently, poor quality of life [4,5], poor sleep quality [6], effect on the biomechanics of movements [7], decreased mobility, muscular strength, flexibility, cardiorespiratory endurance, and the ability to perform common activities of daily living [7,8,9,10,11,12]. In this context, the practice of physical activity promotes favorable physiological adaptations that result in benefits in several of these clinical conditions associated with MSy [13,14,15]. Physical exercise, therefore, should be encouraged, as long as individuals are able to perform.

However, authors have described the difficulty of individuals with MSy to perform physical exercises regularly and maintain adherence to a conventional physical exercise program, due to increased body mass, musculoskeletal limitations, motor deficiencies, reduced physical fitness associated with lack of time, and lack of motivation [13,14,16]. Among the different exercise modalities, the systemic vibration therapy (SVT), that generates the known whole-body vibration exercise (WBVE) has gained progressive popularity, being a strategy, useful, safe, easily practicable, readily accessible, having a low perception of effort, and being well accepted by the individuals [17,18]. WBVE involves exposing individuals to mechanical vibrations that are produced in a vibrating platform (VP). These vibrations are transmitted to the body of the individual which is in contact with the base of the VP [19].

Studies suggest WBVE can be a useful and suitable intervention for management of MSy individuals. WBVE, as an SVT, enhances the strength, improves quality of life, sleep, and flexibility, promotes changes in body composition, reduces pain level, and improves functional and physiological parameters [1,9,20,21,22,23,24,25,26]. Moreover, there is not a systematic review about the effects of WBVE in MSy individuals. Considering this rationale, the aim is to present a systematic review and meta-analysis that analyses the effects of WBVE on individuals with MSy.

## 2. Materials and Methods

The systematic review and meta-analysis were based on the Preferred Reporting Items for Systematic Reviews and Meta-Analysis guidelines [27,28] and the AMSTAR (https://amstar.ca/About_Amstar.php accessed on 3 October 2022), a measurement tool to assess the methodological quality of systematic reviews [29]. The methods were prespecified in a protocol with the PROSPERO International Prospective Register of Systematic Reviews (CRD 42020187319).

### 2.1. Data Sources

An electronic search was conducted in PubMed, Embase, Scopus, Web of Science, ScienceDirect, PEDro, and CINAHL databases on 3 October 2022 and updated on 29 December 2022, using the following search string (“whole-body vibration” OR “whole body vibration”) AND (“metabolic syndrome”). The keywords used in the search were defined based on the PICOS strategy, focusing on individuals with MSy (Participants) receiving WBVE intervention (Intervention) without restrictions regarding comparisons (Comparison), allowing comparisons to placebo, usual care, or control/no intervention. All reported outcomes namely related quality of the life, functionality, pain level, flexibility, cardiovascular responses (blood pressure and heart rate), neuromuscular activation, quality of sleep, rating of perceived exertion, and body composition (Outcomes) were allowed if considered relevant to the studied population (Study design) and were related “randomized clinical trials” and “pseudo-randomized clinical trials” [30].

### 2.2. Eligibility Criteria

**Figure 1:**
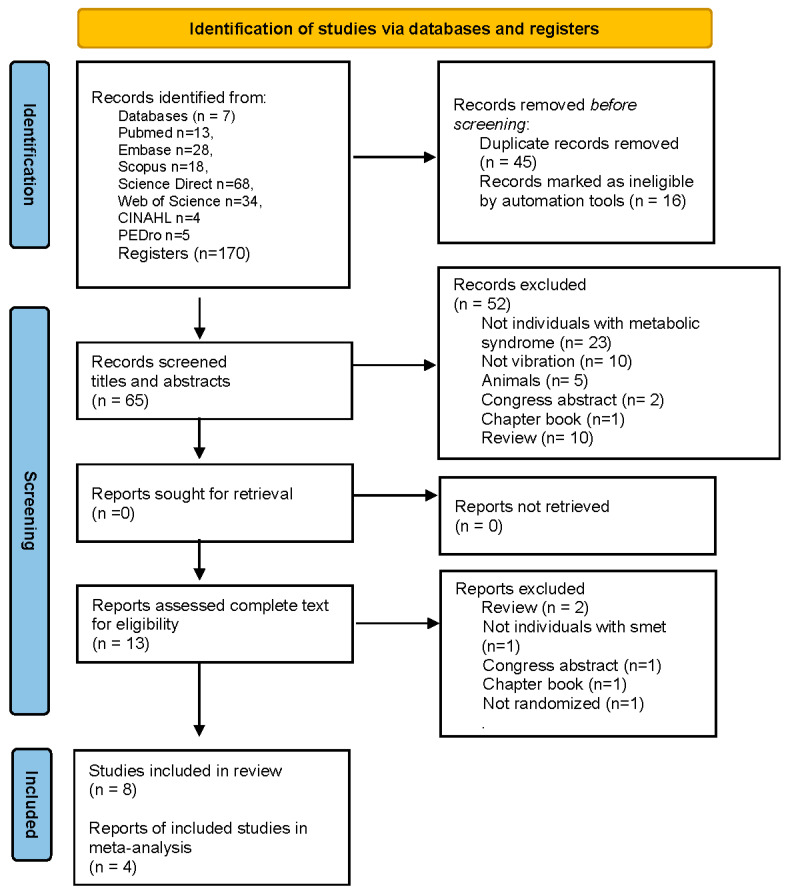
PRISMA flow diagram of the literature selection process.

Inclusion criteria: To be included in this review, the works had to meet the search criteria namely complete articles, in the English language, independent of the year of publication, randomized clinical trials (RCT) or pseudo-randomized clinical trials, and investigative effects of WBVE on MSy individuals. A flowchart (Figure 1), based on the PRISMA analysis was carried out to show the steps in the selection of the full papers analyzed in this review [27].

Exclusion criteria: Exclusion criteria allowed the elimination of unnecessary publications. Papers were excluded if the following applied: (i) published in a language other than English; (ii) with findings not related to Msy; (iii) being replies, editorials, abstracts, congress abstracts, reviews, chapter books, exploratory studies; and (iv) having been conducted with animal, or with other techniques than WBVE, or with combined techniques, or with an occupational approach.

### 2.3. Study Selection

All references were exported to a data management software (EndNote X9), and duplicates were removed. The review was conducted following four steps. Records were identified through database search and reference screening (Identification) and two reviewers (A.C.C., B.B.) independently examined titles and abstracts while irrelevant studies were excluded based on eligibility criteria (Screening). Relevant full texts were analyzed for eligibility (Eligibility) by two reviewers (A.C.C., B.B.) independently, and all relevant works were included in the systematic review (Selection). The disagreement was resolved by a third reviewer (D.C.).

### 2.4. Data Collection Process and Data Items

The same researchers were responsible for data extraction from the included studies. Data regarding study information (author and year), study design, subjects (sample size), demographics (age, sex, body mass index), objectives, WBVE intervention, WBVE protocols, and outcomes were extracted.

### 2.5. Level of Evidence of the Selected Publications

The level of evidence of each selected publication was individually assessed by using the National Health and Medical Research Council hierarchy of evidence [31]. The level of evidence was defined as follows: (i) I, systematic review of level II studies; (ii) II, the RCT; (iii) III-1, pseudo-randomized controlled trial (alternate allocation, as a crossover study or some other similar method); (iv) III-2, comparative study with concurrent controls (non-randomized experimental trial, cohort study, case control study, interrupted time series with a control group); (v) III-3, comparative study without concurrent control (historical control, two or more single arm study, interrupted time series without a parallel control group; (vi) IV, case series with either post-test or pretest/post-test outcomes.

### 2.6. Methodological Quality and Risk of Bias

The methodological quality of the selected clinical trial studies was determined by the PEDRo scale (http://www.pedro.org.au/english/downloads/pedro-scale/, accessed on 10 November 2022). The PEDRo scale consists of eleven items. One item on the PEDRo scale (eligibility criteria) is related to external validity and is generally not used to calculate the method score, leaving a score ranging between 0 to 10. The selected articles with a score of seven or greater in the PEDRo scale were considered of ‘high’ methodological quality, those with a score of five to six of ‘fair’ quality, and with a score of four or below of ‘poor’ quality [32,33]. The Cochrane Collaboration’s tool was used to assess the risk of bias of the included articles [34]. For all the assessments, each manuscript was assigned to one reviewer, cross-checked by a second reviewer and, in case of disagreement, a third reviewer was consulted, and the issue discussed until consensus was reached.

### 2.7. Statistical Analysis

The meta-analysis and quantitative analysis were performed by using Review Manager (v. 5.3) (Cochrane Community, London, UK). All continuous data obtained from the articles were included in this meta-analysis. The mean difference (MD) and 95% confidence interval (CI) were used to analyze the studies. The calculations were performed using a fixed effects and random-effects model. If the trial was a randomized controlled trial, data were extracted from all relevant experimental intervention groups (whole-body vibration training vs. control group). Furthermore, *p* < 0.05 was considered statistically significant. Heterogeneity among the studies was examined by Cochran’s Q and I^2^ statistics, in which values greater than 50% were considered indicative of high heterogeneity [35], and the random-effects model was chosen.

## 3. Results

### 3.1. Results of the Systematic Review

A total of 171 publications was identified through searches in databases and in the hand search and, after the removal of duplicates and paper ineligibles; 70 studies were identified. During the screening process, 57 publications were excluded for not being related to the research question and the complete text of 13 works was reviewed in detail. After careful analysis, six studies were excluded (one was published in a book, two were review articles, one was an abstract, one was not on individuals with MSy, and one was an exploratory study). Finally, eight articles were included in the systematic review and four were included in the meta-analysis. The selection process is summarized in Figure 1.

**Figure 2:**
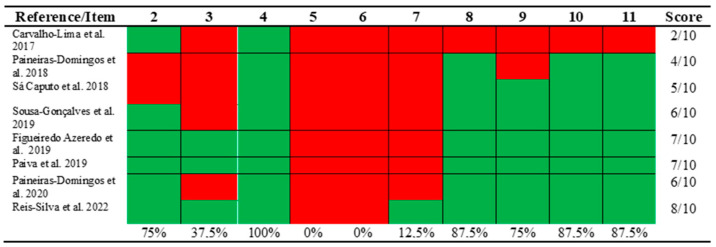
Assessment about the methodological quality with the PEDRo Scale [1,9,20,21,22,23,24,26].

The included publications had a mean score of 5.6, assessing the methodological quality with the PEDro scale (Figure 2), with a minimum of 2 points and a maximum of 8, evidencing high methodological quality. Only three studies [21,24,26] were performed where the allocation was concealed, and due to the nature of the intervention, no studies had participants blinded to group allocation, and there was no blinding of all therapists. Only one study had blinding of all assessors [26]. In this way, three studies [21,24,26] were considered of “high” quality, three [1,9,23] of “fair” quality, and two [20,22] of “poor” quality.

**Figure 3:**
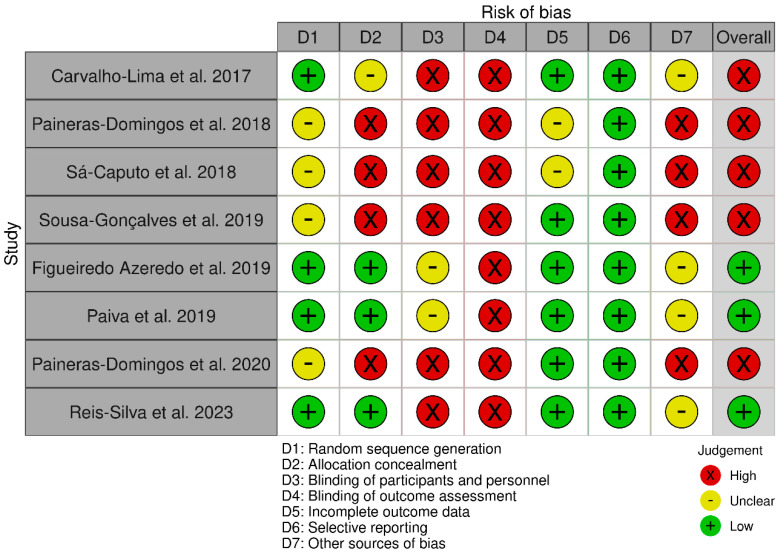

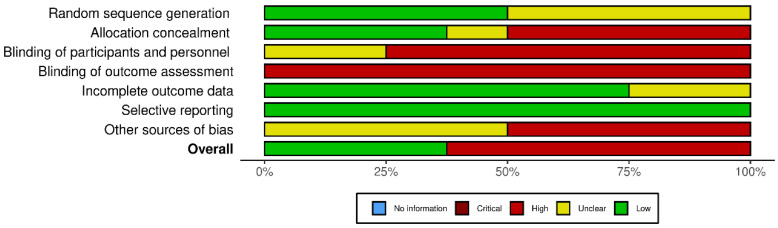
Risk of bias summary: authors assessment for each risk of bias criterion [1,9,20,21,22,23,24,26].

The risk of bias of included studies was assessed used the Cochrane risk of bias tool (Figure 3). Of the studies included in this systematic review, overall, only three studies [21,24,26] were considered to be at “low” risk of bias and five studies [1,9,20,22,23] were considered to be at “high” risk of bias.

Table 1 shows the characteristics of the articles and participants, the aims of the studies, and the outcomes of the selected articles with the level of evidence of the selected papers. The level of evidence (NHMRC, 2003–2007) [31] of the four studies included in the current review [20,21,24,26] was considered LE II and four studies [1,9,22,23] were considered LE III-1.

Several outcomes were assessed: (i) quality of the life through the World Health Organization Quality of Life-Bref Questionnaire (WHOQOL-BREF) [20]; (ii) functionality through the Short Physical Performance Battery (SPPB) [22]; (iii) pain level, trunk flexibility, and cardiovascular responses (blood pressure and heart rate) [9]; (iv) neuromuscular activation using the surface electromyography pattern (root mean square (RMS)) of the vastus lateralis muscle and the ROM of the knees [1]; (v) quality of sleep through the Pittsburgh Sleep Quality Index, Epworth Sleepiness Scale, and Berlin Questionnaire [21]; (vi) flexibility through anterior trunk flexion and Rating of Perceived Exertion (RPE) [24]; (vii) chronic pain level by the Numeric Rating Scale [23]; (viii) body composition (to estimate total, and regional body fat mass, total and regional lean mass, total body water, skeletal muscle mass, and bone mineral content) was analyzed using a Tetrapolar Electrical Bioimpedance Analyzer (In Body 370, BIOSPACE, Seoul, Republic of Korea) and the anthropometric characteristics were determined through waist circumference (WC), neck circumference (NC), and waist-hip ratio (WHR) [26].

Regarding the main findings, Table 1 shows that WBVE showed the following effects: improved the quality of life in different domains of the WHOQOL-BREF [20,23]; advanced some functional parameters such as balance, gait, and lower limb strength [22]; induced physiologic changes contributing to decrease pain level [9,23], to maintain cardiovascular responses [9] as well as to increase the flexibility [9,24]; induced changes in neuromuscular activation [1] and increased ROM of the knees [1]; interfered with physiological mechanisms leading to the improvement of the quality of sleep [21]; declined the RPE [24]; made changes in body composition such as a decrease in WC, a decrease in segmental fat in the left arm, right arm, and trunk, and an increase in bone content [26]. In general, the sample size was small, ranging between 19 and 44 participants.

Detailed descriptions of the WBVE protocols (i.e., frequency, amplitude, peak to peak displacement, position of the individual in the base of the platform, type of platform) are reported in Table 2. Considering the biomechanical parameters, the range of the frequency of the mechanical vibrations varied from 5 up to 16 Hz; one work used only 5 Hz [9], four used 5 up to 14 Hz [1,20,22,23], and only three articles used 5 up to 16 Hz [21,24,26], while the peak-to-peak displacements were 2.5, 5.0, and 7.5 mm. In general, the majority of participants were seated on a chair in front of the platform with their hands kept on their knees and the feet positioned on the base of the platform and/or stood on the base of the platform in a squat position (knees flexed at 130°) varying between squats static or static and dynamic. The WBVE intervention protocols were homogenous; the working time in each position was 1 min followed by 1 min of rest, however, the intervention varied in training frequency, number of repetitions, number of exercises per session, and session duration.

### 3.2. Results of the Meta-Analysis

Three meta-analyses were performed of four included studies, which reported results on the quality of life, pain level, and flexibility in the different intervention groups.

**Figure 4:**
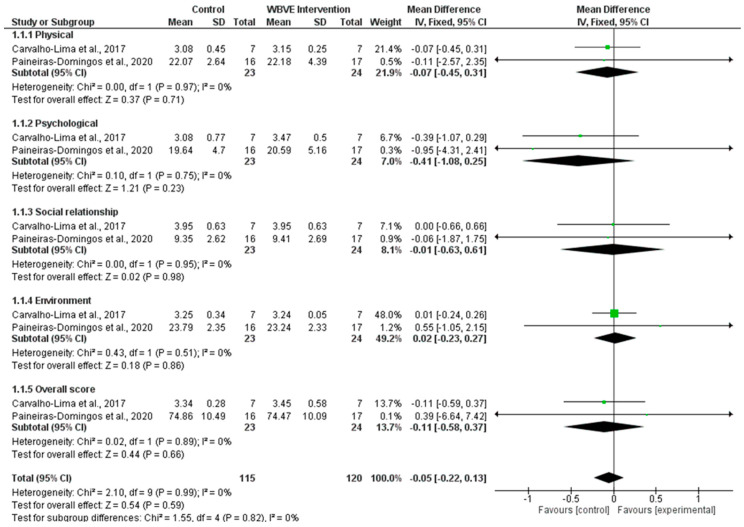
Results of the meta-analysis for the effects of WBVE on quality of life [20,23].

Quality of Life: No significant changes were observed on the overall score concerning the WHOQOL-BREF (*n* = 335 participants, MD = −0.05; 95% CI [−0.22, 0.13]; *p* = 0.59; heterogeneity: *p* = 0.99, I^2^ = 0%) as well as on the physical, psychological, social relationship, and environment domains for the quality of life in the MSy individuals after the intervention period in either the protocols, control or the WBVE, while the differences between the interventions were not statistically significant (Figure 4).

**Figure 5:**
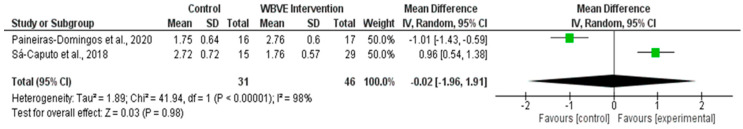
Results of the meta-analysis for the effects of WBVE on pain level [9,23].

Pain Level: No significant changes were observed on a scale of 0 to 10 after the intervention period in either the FF or the VF, and the differences between the interventions were not statistically significant (*n* = 77; MD = −0.02; 95% CI [−1.96, 1.91]; *p* = 0.98; heterogeneity: T^2^ = 1.89; *p* < 0.00001; I^2^ = 98%) (Figure 5). 

**Figure 6:**
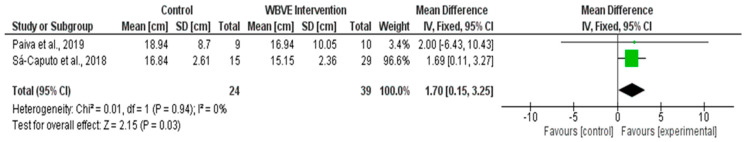
Results of the meta-analysis for the effects of WBVE on flexibility [9,24].

Flexibility: As shown in Figure 6, for the FAT test (*n* = 63 participants, MD = 1.70; 95% CI [0.15, 3.25]; *p* = 0.03; heterogeneity: *p* = 0.94, I^2^ = 0%) showed a significantly improved flexibility with reduced middle finger distance to the ground after the intervention period, for the group that performed systemic vibration therapy over all the exposure time, and did not present any change in flexibility for the control or minimal intervention group.

## 4. Discussion

The main goal of this systematic review and meta-analysis was to assess the effects of WBVE in MSy individuals. After analyzing the included studies, the results suggest that WBVE may be a valid and feasible intervention in this population. Nonetheless, according to the risk of bias, the included clinical trials still lack detailed information on the methods used and have methodological errors that compromise their internal validity. The methodological quality of the included studies was moderate, mainly regarding the concealed allocation, the lack of blinded subjects, the therapy administrators, and the referred assessors in outcome measurement.

Effects in biomechanics, range of motion, motor neuron and sleep quality were reported. However, the limited number of studies addressing each of the outcomes limits the ability to establish its relevance to clinical practice. Further additional studies with high methodological quality are needed to address these effects in the future and to allow solid recommendations to be established.

Effects of Positioning in WBVE: The studies presented two types of position, sitting in the chair with feet on the platform, and/or standing on the platform in the squat position, both positions providing significant gains in all measured variables. Two studies [21,24] provided static and dynamic squats, four works [1,20,22,23] provided only static squats, while no squat was envisaged by Sá-Caputo et al. [9]. Although vibration is transmitted to the whole body in all postures [36], it is still unclear what is the best positioning to be adopted for this population. 

Effects on Functionality: One study [22] reported significant functional improvements in the WBVE groups after 10 sessions training on the vibrating platform with a comparable progressive and increased frequency, and promoting an additional effect on local stochastic muscle endurance, corroborating with parameters and results of Sá-Caputo et al. [25]. This suggests that this exercise shows a better performance and can be offered to these individuals, contributing to what is advocated as intervention strategy in this syndrome—a change to a better lifestyle [22].

Effects on Quality of Life: Two studies [20,23] identified benefits in different domains on quality of life with similar WBVE intervention protocols (intensity, training frequency, number of repetitions, number of exercises per session, session duration, and position suggesting the effectiveness of the protocol with a progressive and increased frequency over 6 weeks (one or two times per week). This finding is in line with recent studies showing that WBVE intervention is able to improve the quality of life in elderly adults [37] and chronic conditions [38]. Nevertheless, despite the observed benefits, little is known about which is the best intervention protocol with WBVE, for the quality of life in general for these individuals, as demonstrated by the results of the meta-analysis.

Effects on Physiological Parameters: The WBVE intervention was capable of exerting positive effects on blood pressure [9], heart rate [9,21] and subjective perception of effort [24], even using different WBVE protocols (intensity, training frequency, position on the vibrating platform, time duration) in three of the included publications [9,21,24]. Furthermore, a systematic review of Rubio-Arias et al. [39] also observed that WBV training could be an effective training modality to reduce blood pressure (clinically relevant) and resting heart rate. By contrast, no effects were observed by Sá-Caputo et al. [25]. In this context, in general, WBVE was presented as a safe exercise considering the clinical physiological aspects, helping to maintain cardiovascular responses, and not promoting exacerbations that contribute to reducing the cardiovascular risk factor. 

Effects on Flexibility: Two studies [9,24] reported an improvement of trunk flexibility, suggesting good vertebral function, observed both after acute and chronic WBVE exposure. In this respect, WBVE could be useful to aid patients in the rehabilitation of physical ability and good physical health [38,40]. These results, such as the meta-analysis, suggest benefits that favor the effect of intervention with vibratory-systemic therapy, regardless of the stimulus magnitude (frequency, intensity, and acceleration), provided there is vibration throughout the full time of intervention. This fact corroborates with the finding that exercise promotes warm-up and consequent increase in flexibility [41].

Effects on Pain Level: Due to the pain or even the low physical fitness, most individuals with MSy are unable or unwilling to perform exercises [40]. WBVE demonstrated a statistically significant decrease in the pain level with different intervention protocols in two articles [9,23] of this review. These results point to a positive effect even after a single trial of WBVE. Moreover, in this modality, the mechanical vibration stimuli, according to studies can affect central mechanisms, cortical reorganization [42], and second-order nociceptive activities [43], thereby reducing pain, as well as being associated with lower chronic musculoskeletal pain [44], lower neuropathic pain symptoms, and reduced acute and chronic pain in chronic diseases [45]. However, when quantitatively comparing the different types of intervention and protocols, according to the meta-analysis, there is still a gap regarding the better and more efficient intervention protocol for the pain variable between studies [9,23], for these MSy individuals.

Effects on Body Composition: Reis-Silva et al. [26] suggest changes in the body composition (WC, in segmental fat mass and bone content) in these MSy individuals after the WBVE, and these findings are in agreement with a systematic review by Alavinia et al. [46] that indicates a positive effect of WBV therapy on reducing fat mass (%/kg), especially when combined with conventional body mass loss interventions; specifically, diet and exercise in obese adults. In contrast, a recent systematic review of Rubio-Arias et al. [39], did not observe significant improvements in variables associated with body composition and metabolic syndrome. However, the cited reviews and the present review, all suggest, that modality exercise therapy (WBVE) can be capable of contributing to improvements in metabolic health and in reducing the cardiovascular risk factor.

### 4.1. Strengths

The strength of this work is related to the identification of a non-pharmacological, safe, well acceptable, and not expensive intervention (i.e., WBVE) which is able to establish improvements of several clinical parameters in MSy individuals.

### 4.2. Study Limitations

The findings of this systematic review and meta-analysis must be however interpreted with a fair degree of caution. One of the limitations is that it is not possible to draw convincing conclusions based on a small number of relevant studies (only eight publications) evaluated the intervention with WBVE in MSy individuals and most of the papers were for single research studies. Further, it is difficult to provide strong evidence of the effects of WBVE on the outcomes, due to the differences existing in the examined parameters and intervention protocols (frequency, amplitude, position of the individual in the base of the platform, type of platform). Moreover, only papers in the English language were accessed.

## 5. Conclusions

WBVE interventions in MSy individuals evidenced good patient compliance. This intervention exerts a positive impact on pain level, electromyographic activity, cardiovascular responses, quality of life, flexibility, mobility, range of knee motion, sleep quality, functionality, and body composition. Therefore, implementing WBVE interventions (i.e., tailoring the protocols according to the biochemical, functional, and clinical characteristics of a single patient) in this population could be recommended in order to improve the parameters further. In this respect, WBVE effects should be better investigated and further additional studies with high quality randomized clinical trials, proper allocation of concealment and blinding, are needed in order to establish the most adequate WBVE intervention protocols for this population.

Future studies are required to investigate WBVE effects, comparing the effects of different types of WBVE (synchronous and side alternating) and different vibration frequencies and amplitudes. Furthermore, the study of the age-dependent and gender-related effects of WBVE in MSy individuals will require careful consideration in order to adapt this treatment to the specific need of a single patient. More than with other physical exercises, optimization of the WBVE-induced results will depend on single patient-tailored treatment.

## Figures and Tables

**Table 1:** Summary table of the included studies in the review with main findings.

Study	Study Design	Country	Demographics	Aim	Results	Level of Evidence
Carvalho-Lima et al., 2017 [20]	A Randomized Clinical Trial	Brazil/Spain	*n* = 21 (7 men/14 women) (i) CG: *n* = 7; (ii) WBVE1: *n* = 7; (iii) WBVE2: *n* = 7; Age 66.65 ± 2.90 years.	Evaluate the effect of WBVE on the quality of the life of individuals with MSy through the WHOQOL-BREF questionnaire.	WBVE in a protocol (one or two times per week) with a progressive and increased frequency improves the quality of life of patients with MSy in different domains of the WHOQOL-BREF.	II
Paineiras-Domingos et al., 2018 [22]	Pseudo-randomized controlled trial study	Brazil/Italy	*n* = 39 (i) WBVEeG: *n* = 22 (ii) CG: *n* = 17; Age 47 to 69 years.	Evaluate functionality through SPPB in individuals with MSy after WBVE.	WBVE is a feasible physical activity for individuals with Msy. Through the adhesion to this type of aerobic activity, some functional parameters such as balance, gait, and lower limb strength are improved. Consequently, a better quality of life can be offered to these individuals, contributing to what is advocated as intervention strategy in this syndrome: a change to a better lifestyle.	III-1
Sá-Caputo et al., 2018 [9]	Pseudo-randomized controlled Trial	Brazil/France/Spain/Australia	*n* = 44 (i) CG: *n* = 15 Age 58.20 ± 9.11 years BM 87.43 ± 18.02 kg (ii) WBVE exercise: *n* = 29 Age 61.10 ± 8.39 years, BM 83.65 ± 16.27 kg	Assess the acute effect of WBVE exercise, with low frequency (5 Hz), on the pain level, trunk flexibility, and cardiovascular responses ([BP] and [HR]) in individuals with Msy.	WBVE exposure with low frequency (5 Hz) is responsible in inducing physiological parameters that contribute to decrease the PL and to increase the flexibility as well as to maintain cardiovascular responses (HR and BP) in individuals with Msy.	III-1
Sousa-Gonçalves et al., 2019 [1]	Quasi-randomized and cross-over controlled trial study	Brazil/France/Italy	*n* = 39 (i) CG: *n* = 17 Age 58.1 ± 2.07 years, BM 88.8 ± 4.08 kg (ii) TG: *n* = 22 Age 60.7 ± 1.91 years, BM:83.1 ± 3.65 kg.	Analyze the effects of WBVE on Msy individuals’ neuromuscular activation using the sEMG pattern RMS of the VL muscle and on the ROM of the knees.	WBVE can be a modality of exercise to increase the neuromuscular activity of the VL muscle using a 5-week protocol. An increase in ROM of the knees in individuals with Msy was not observed with the same protocol. WBVE appears to be an adequate strategy to improve neuromuscular activity in individuals with Msy, overweight, and obesity, being a potential opportunity for the management of physical impairment in these individuals. Nevertheless, further additional studies with larger samples and more prolonged periods of WBVE exposure are needed to confirm our preliminary findings.	III-1
Figueiredo-Azeredo et al., 2019 [21]	Cross-sectional and randomized study	Brazil//France/Italy	*n* = 19 (i) GFF: *n* = 9 (ii) GVF: *n* = 10 Age 58.79 ± 12.55 years Height: 1.62 ± 0.09 m BM: 86.27 ± 15.03 kg	Investigate the effect of WBVE exercise on parameters related to the sleep quality in MSy individuals.	WBVE intervention was capable of interfering with physiological mechanisms with effects on the WC and HR, leading to the improvement of the quality of sleep in MSy individuals. WBVE exercise might be an important clinical intervention for the management of some factors associated with poor quality of sleep (FFG and VFG) and in the daytime sleepiness in MSy individuals with variable frequencies (5–16 Hz).	II
Paiva et al., 2019 [24]	Randomized clinical trial pilot study	Brazil/France/Italy/New Zealand	*n* = 19 patients Age 58.79 ± 12.55 years Height: 1.62 ± 0.09 m BM: 86.27 ± 15.03 kg	Evaluate the acute and cumulative effects from 6 weeks of WBVE exercise using 2 biomechanical conditions [FF] and [VF]) on flexibility and RPE in MSy individuals.	WBVE exercise improved the flexibility and decreased the RPE in MSy individuals. These findings suggest that WBVE exercise can be incorporated into physical activities for MSy individuals.	II
Paineiras-Domingos et al., 2020 [23]	Pseudo-randomized crossover study	Brazil/France/Italy	*n* = 33 patients (i) WBVEeG, *n* = 17, (15 women/02 men) Age 61.1 ± 8.4 years (ii) CG, *n* = 16,(14 women/02 men) Age 58.2 ± 9.1 years	Evaluate the effects of WBVE on quality of life and chronic pain in individuals MSy.	WBVE in MSy individuals is capable significantly (i) to promote na improvement of QoL considering the physical and psychological domains, as accumulative effect and (ii) to reduce CPL in the acute interventions in the first and in the last sessions. Therefore, WBVE would represent a suitable and useful physical activity that could be included in health programs for MSy individuals, following the WHO recommendations.	III-1
Reis-Silva et al., 2022 [26]	Randomized controlled trial	Brazil/France/Italy	*n* = 22 patients (16 women/6 men), FFG-WBVE (*n* = 12; median age = 50.50 years and BMI = 31.95 kg/m^2^) VFG-WBVE (*n* = 10; median age = 57.50 years and BMI = 32.50 kg/m^2^)	Investigate the effects of two 6-week WBVE protocols on body composition in patients with MSy.	6-weeks of VFG-WBVE, performed actively, can positively modify body composition in individuals with MSy. The improvement in fat mass, on the left and right arms and trunk, is clinically noteworthy, since the reduction of this fat tissue can contribute to improvements in metabolic health and reduce the cardiovascular risk factor.	II

MSy: metabolic syndrome; WBVE: whole-body vibration exercise; VFG-WBVE/GVF/VF: group variable frequency; FFG-WBVE/GFF/FF: group fixed frequency; CG: control group; BMI: body mass index; BM: body mass; CPL: chronic pain level; WHO: World Health Organization; QoL: quality of life; RPE: Rating of Perceived Exertion; sEMG: surface electromyography; RMS: root mean square; VL: vastus lateralis muscle, ROM: range of motion; BP: blood pressure; HR: heart rate; SPPB: short physical performance battery.

**Table 2:** Protocol intervention WBVE with studies in the review.

Study	WBVE Intervention	Parameters	Type of Vibrating Platform	Positioning	Time WBVE
Carvalho-Lima et al., 2017 [20]	10 weeks WBVE1 one time per week; WBVE2 twice times per week.	CG: platform turn off; WBVE1/WBVE2: Peak-to-peak displacements of (2.5, 5.0, and 7.5 mm), frequency progressively 5 Hz–14 Hz.	Side Alternating vibrating Platform. (Novaplate Fitness Evolution, DAF Produtos Hospitalares Ltd.a., São Paulo, Brazil)	1 session/week: sat in a chair placed in in front of the platform with flexion of the knees. Their feet were on the platform base in three positions. (5 Hz). 2–10 session/week: squat position and the frequency was progressively increased by one unit for each session (6 Hz–14 Hz).	The work time was 1 min with 1 min rest in each peak-to-peak. This sequence was repeated two more times in each session.
Paineiras-Domingos et al., 2018 [22]	10 sessions	CG: platform turn off. WBVEeG: Peak-to-peak displacements of 2.5, 5.0, and 7.5 mm, frequencies ranged from 5 up to 14 Hz.	Side Alternating vibrating Platform. (Novaplate Fitness Evolution, DAF Produtos Hospitalares Ltd.a., São Paulo, Brazil)	1 session: seated on a chair in front of the platform with hands kept on their knees and feet positioned on the base of the platform. (5 Hz). 2–10 session: standing on the base of the platform with a squat, static position (knees flexed at 130°). (6–14 Hz).	The work time was 1 min with 1 min rest in each peak-to-peak. (3 min in each amplitude, with a resting period of 1 min) (total time: 18 min/session)
Sá-Caputo et al., 2018 [9]	1 session	CG: platform turn off. WBVE exercise group: Peak-to-peak displacements of 2.5, 5.0, and 7.5 mm, frequency 5 Hz and corresponding to peak accelerations of 0.12, 0.25, and 0.35 g, respectively.	Side Alternating vibrating Platform. (Novaplate Fitness Evolution, DAF Produtos Hospitalares Ltd.a., São Paulo, Brazil)	Sitting in a chair with feet on the platform with knees flexed.	3 bouts (1 min each). The session consisted of 9-min bout of work interspersed with 1-min passive rest period between each bout (total time: 17 min).
Sousa-Gonçalves et al., 2019 [1]	5 weeks (twice a week) 10 sessions	CG: platform turn off.; TG: Peak to peak displacements of 2.5, 5.0, and 7.5 mm) and with a frequency of 5–14 Hz.	Side Alternating vibrating Platform. (Novaplate Fitness Evolution, DAF Produtos Hospitalares Ltd.a., São Paulo, Brazil)	1 session: Seated on a chair placed in front of the platform with a 130° knee flexion. Their feet, shoeless, were placed on the base of the platform, alternately in three positions. 2–10 session: Standing on the base of the platform in a squat, static position (130° knee flexion), and the frequency was progressively increased by 1 unit per session. (6–14 Hz).	The working time in each position was 1 min followed by 1 min of rest. This sequence was performed three times.
Figueiredo-Azeredo et al., 2019 [21]	6 weeks (twice a week)	FFG: Peak to peak displacements of 2.5, 5.0, and 7.5 mm) and with a frequency of 5 Hz. WBVE Group: Peak to peak displacements of 2.5, 5.0, and 7.5 mm) and with a frequency of 5–16 Hz.	Side Alternating vibrating Platform. (Novaplate Fitness Evolution, DAF Produtos Hospitalares Ltd.a., São Paulo, Brazil)	Squat position, barefoot and with (130° knee flexion). Dynamic and static squats interspersed sessions.	FFG performed 60 s of vibration (10 s of vibration and 110 s of non-vibration) and 60 s of non-vibration in each bout and WBVE Group performed 60 s of vibration and 60 s of non-vibration in each bout. From 1 to 4 weeks were performed 3 bouts in each session, (total time:18 min); From 5 to 8 weeks, were performed 4 bouts in each session (total time: 24 min); From 9 to 12 weeks were performed 5 bouts in each session, (total time:30 min).
Paiva et al., 2019 [24]	6 weeks (twice a week) 12 sessions	FF-WBVE: Peak to peak displacements of 2.5, 5.0, and 7.5 mm) and with a frequency of 5 Hz. VF-WBVE: Peak to peak displacements of 2.5, 5.0, and 7.5 mm) and with a frequency of 5–16 Hz.	Side Alternating vibrating Platform. (Novaplate Fitness Evolution, DAF Produtos Hospitalares Ltd.a., São Paulo, Brazil)	Dynamic and static squats interspersed sessions (start 1st with static squat).	FF-WBVE: 10 s plus 50 s with the vibration machine turned off (1 min) in the squat position and rested for 1 min. Performed 3 times in the first and second weeks (total time:18 min). In the third and fourth weeks, the sequence was performed 4 times (total time:24 min) and in the fifth and sixth weeks, 5 times (total time: 30 min). VF-WBVE: 1 min with the vibration machine turned on and rested for 1 min. Performed 3 times in the first week (total time of 18 min), 5 Hz (1th session), and 6 Hz (2th session); in the second week (total time:18 min), 7 Hz (3th session) and 8 Hz (4th session); in the third week (total time: 24 min), 9 Hz (5th session) and 10 Hz (6th session); in the fourth week (total time:24 min), 11 Hz (7th session) and 12 Hz (8th session); in the fifth week (total time:30 min), 13 Hz (9th session) and 14 Hz (10th session); and in the sixth week (total time: 30 min), 15 Hz (11th session) and 16 Hz (12th session).
Paineiras-Domingos et al., 2020 [23]	10 weeks 10 sessions	CG: platform turn off. WBVEeG: Peak to peak displacements of 2.5, 5.0, and 7.5 mm) and with a frequency of 5–14 Hz.	Side Alternating vibrating Platform. (Novaplate Fitness Evolution, DAF Produtos Hospitalares Ltd.a., São Paulo, Brazil)	1 session: Seated on a chair placed in front of the platform with a 130° knee flexion. Their feet, shoeless, were placed on the base of the platform, alternately in three positions. 2–10 session: Standing on the base of the platform in a squat, static position (130° knee flexion), and the frequency was progressively increased by 1 unit per session. (6–14 Hz).	Work time was 1 min and 1 min rest in each position. This procedure was done three times with the total time WBVE exercise: 18 min.
Reis-Silva et al., 2022 [26]	6 weeks, twice a week (12 sessions)	FFG-WBVE: Peak to peak displacements of 2.5, 5.0, and 7.5 mm) and with a frequency 5 Hz in all sessions. VFG-WBVE: Peak to peak displacements of 2.5, 5.0, and 7.5 mm) and with a frequency of 5–16 Hz with an increase of 1 Hz at each session.	Side Alternating vibrating Platform. (Novaplate Fitness Evolution, DAF Produtos Hospitalares Ltd.a., São Paulo, Brazil)	The patient was positioned barefoot, in a semi-squat position with 130° knee flexion controlled by a goniometer, trunk erect, hands lightly resting on the side bar of the platform, relaxed shoulders, and head in a neutral position. The WBVE was performed statically or dynamically (in alternating sessions).	FFG-WBVE: the individual performed 1 min of WBVE (10 s with vibration and 50 s without vibration- semi squat position) and 1 min of rest) in 2.5, 5.0 and 7.5 mm PPD. This sequence was performed 3 times from the 1st to the 4th session (18 min of total time); 4 times from the 5th to the 8th session (24 min of total time) and 5 times from the 9 to the 12th session (30 min of total time). VFG-WBVE: the individual performed 1 min of WBVE (semi squat position) and 1 min of rest in 2.5-, 5.0- and 7.5-mm PPD. This sequence was performed 3 times from the 1st to the 4th session (18 min of total time); 4 times from the 5th to the 8th session (24 min of total time) and 5 times from the 9 to the 12th session (30 min of total time).

WBVE: whole body vibration exercise; VFG-WBVE/GVF/VF/WBVE Group/WBVEeG: group variable frequency; FFG-WBVE/GFF/FF: group fixed frequency; CG: control group; TG: treatment group; PPD: peak to peak displacements.

## Data Availability

Not applicable.
